# Fish Reproductive Biology and Embryogenesis

**DOI:** 10.3390/ani16193015

**Published:** 2026-09-25

**Authors:** Renato Massaaki Honji, Amanda da Silveira Guerreiro

**Affiliations:** 1 Laboratório de Aquicultura e Ecofisiologia Marinha (LAQUEFIM), Departamento de Fisiologia, Instituto de Biociências, Universidade de São Paulo (IB/USP), Rua do Matão, Travessa 14, No. 321, São Paulo 05508-090, SP, Brazil; 2 Departamento de Fisiologia, Instituto de Ciências Biológicas (ICB), Universidade Federal do Rio Grande (FURG), Av. Itália s/n km 8, Campus Carreiros, Rio Grande 96203-900, RS, Brazil

## 1. Introduction

Over millions of years of evolution, fish have developed a diverse array of reproductive strategies including many alternative physiological, morphological, and behavioral tactics, as well as distinct patterns of embryonic and larval development, which have allowed them to successfully reproduce and survive [1,2]. Moreover, the variety of ecological habitats and conditions they face represents a central component of their capacity to physiologically adjust and thrive [3,4]. Reproductive success therefore depends on the coordinated action of processes encompassing gamete production, sexual maturation, fertilization, embryogenesis, hatching, and early larval development [5]. These processes may involve (a) external or internal fertilization, although external fertilization is the predominant reproductive strategy among most species; (b) nest, pelagic or benthic/demersal spawning; (c) highly specialized mating systems such as spawning aggregations; (d) parental care; (e) reproductive migrations, as observed in rheophilic species; and/or (f) other species-specific adaptations that connect physiology and ecology to life-history strategy [2,3,6].

Advances on topics such as neuroendocrinology [7], gametogenesis [8], steroidogenesis [9], developmental biology [10], and molecular physiology [11] have substantially expanded our knowledge on the mechanisms that regulate fish reproduction and embryogenesis. In this context, studies addressing oogenesis, spermatogenesis, sex differentiation and determination, neuroendocrine control, steroidogenesis, reproductive behavior, maternal and paternal effects, and environmental regulation continue to reveal the remarkable plasticity of fish reproductive systems [6,12]. At the same time, embryological research provides detailed information on cleavage, gastrulation, axis formation, organogenesis, morphogenetic processes, epigenetic regulation, and the transition from embryonic to larval life [10,13]. These areas are particularly relevant because reproductive and developmental processes are sensitive to environmental conditions, including temperature, photoperiod, nutrition, water quality, contaminants, and climate-driven changes [8].

The Special Issue *“Fish Reproductive Biology and Embryogenesis”* brings together original studies and critical reviews examining these processes from evolutionary, ecological, physiological, developmental, and molecular biology viewpoints. Instead of discussing reproduction and embryogenesis as isolated fields, this Special Issue exemplifies a biological continuum, in which endocrine signaling, gamete development, spawning, embryonic ontogeny, larval performance, and population-level outcomes are closely interconnected. Additionally, the studies included in this Special Issue highlight the applied relevance of this knowledge for aquaculture, fishery management, and conservation of threatened species. Therefore, the papers assembled in this volume comprise multiple levels of biological organization and a diverse range of teleost species. Together, they provide new insights into endocrine and cellular regulation of reproduction, reproductive seasonality and life-history strategies, manipulation of sexual differentiation, broodstock performance, embryonic development, and the ecological consequences of reproductive plasticity. This scope highlights the importance of integrating mechanistic and comparative approaches when attempting to understand how fish reproduce and how reproductive traits influence persistence under both natural and captive conditions.

## 2. Physiological and Endocrine Regulation of Fish Reproduction

At the physiological level, fish reproduction is controlled by coordinated interactions among the brain–pituitary–gonadal (B-P-G) axis, gonadal development, steroidogenesis, environmental signals, and reproductive behavior. The goldfish, *Carassius auratus*, remains one of the most informative comparative models for understanding these interactions. Building on this, Kobayashi et al. [14] demonstrate that once females are properly exposed to a stimulus, such as rising temperature, the presence of spawning substrate, and likely pheromones, they experience a pre-ovulatory LH (luteinizing hormone) surge that drives steroidogenesis, oocyte maturation, and the release of steroid hormones into water. This is where the review critically expands knowledge: in goldfish reproduction, the hormones and pheromones are essentially one and the same. For instance, the authors show that hormones, like DHP (17,20β-dihydroxy-4-pregnen-3-one) and the prostaglandin F2α, are released into water and act on conspecifics’ physiology and behavior to trigger spawning. Consequently, both sexes have developed highly sensitive olfactory systems to capture steroidal products in the water, making goldfish the animals with the best pheromone system described among fishes. The authors also highlight that the goldfish presents a sexual bipotentiality, despite being a non-sex-changing gonochoristic. This is relevant because it shows that the goldfish brain is not strictly differentiated into a single sex circuit, where sexual bipotentiality can be carried into adulthood. Interestingly, this capacity is more pronounced in males than females, but it remains unclear why the goldfish brain has this characteristic and what biological value this might have. However, this plasticity leaves goldfish susceptible to endocrine-disruption chemicals, which are a growing concern in aquatic ecosystems.

The cellular regulation of gametogenesis is further demonstrated by Ventriglia et al. [15]. By treating pre-pubertal *Argyrosomus regius* fish, also known as meagre, with recombinant follicle-stimulating hormone (rFsh), the authors observed larger testes and advanced spermatogenesis, with wider seminiferous tubules, increased sperm presence in the lumen, and reduced testicular apoptosis in comparison to controls. This contrasts deeply with adult spermatogenesis, which is marked by an increase in apoptotic cells, demonstrating that apoptosis plays opposite roles depending on context. These findings reinforce the point that apoptosis is not simply an indicator of cellular loss but a dynamic component of spermatogenic regulation whose functional significance changes according to reproductive stage.

The understanding of endocrine control can also be valuable in reproductive biotechnology. In many cases, monosex culture, particularly female-only production, is preferred in aquaculture to enhance production, since females usually exhibit faster growth rates, better meat quality, and higher market value. Considering this, Dokkaew et al. [16] fed larvae of the long-whiskered catfish *Mystus gulio* with microencapsulated 17β-estradiol (E_2_) and achieved the highest specific growth rate, along with a feminization success rate of 99%. Reduced testosterone levels alongside increased E_2_ levels were further confirmed, and *cyp19a*, *erb1* and *erb2* mRNA levels were upregulated, demonstrating the system’s ability to generate monosex female populations of *M. gulio*. The study demonstrates how knowledge of the timing and hormonal regulation of sexual differentiation can be applied to monosex production, while simultaneously emphasizing the importance of dose, exposure period, and delivery system in the development of effective and controlled protocols.

## 3. Reproductive Strategies, Spawning Dynamics, and Population Persistence

As mentioned above, reproductive success cannot be interpreted solely from endocrine mechanisms because spawning performance is also influenced by environmental conditions, broodstock history, age, behavior, and species-specific life-history traits. Huanacuni et al. [17] clearly show the effect of multiple variables, such as animal age, nutrition, temperature and captivity itself, on a broodstock population. The authors studied five consecutive spawning seasons of the Peruvian grunt, *Anisotremus scapularis*, a commercially relevant fish species facing overexploitation in natural ecosystems. One of the main findings was that spawning showed marked seasonality from October to May, consequently influenced by temperature and photoperiod, which is key because extreme weather events (such as heat waves) can impact fish reproduction. In addition, the authors observed that the highest reproductive output occurred in the first season and declined in the following years.

At the population level, reproductive traits also provide information for understanding resilience and for developing science-based management measures. For some fish species, habitat degradation and overfishing have led to significant declines in wild populations. Owing to this, Xiang et al. [18] investigated the reproductive biology of the dark sleeper *Odontobutis potamophila*, a species facing exactly this problem. The authors observed that both sexes mature after one year, at body lengths of approximately 73 mm, with spawning occurring from March to June and peaking in May. This demonstrates that *O. potamophila* exhibits a reproductive strategy characterized by early maturation at a small body size and high reproductive output, providing a scientific basis for setting fishery management. The results highlight that science-based measures, like covering the entire reproductive window, establishing an annual fishing ban (from March to June), and adjusting size limits, will aid not only in the conservation of this species, but also in broodstock management for aquaculture.

The ecological importance of reproductive strategies is also demonstrated by Hu et al. [19]. The authors investigated the reproductive biology of two invasive tilapia species, *Coptodon zillii* and *Oreochromis niloticus*, in the Jinghong Reservoir, located in China, to elucidate which mechanisms underlie their invasiveness, coexistence, and success. Results show that the species presented prolonged breeding seasons, lasting from April to September for *C. zillii*, peaking in May and June, and from May to November for *O. niloticus*, peaking in August. In addition, both species are batch spawners, although the eggs from *O. niloticus* are relatively larger in diameter. In contrast, *C. zillii* displayed higher absolute fecundity, length-relative fecundity, and weight-relative fecundity. Because those species reach sexual maturity at small body sizes, this might enable them to enter reproductive season earlier, thereby driving rapid population expansion. Furthermore, the species follow different strategies: *C. zillii* adopted a more R-selected strategy, whereas *O. niloticus* exhibited a more K-selected strategy, which is a clear display of reproductive strategies for colonizing ecosystems. 

## 4. Embryogenesis and Early Development: From Fundamental Biology to Applied Conservation and Aquaculture

Embryogenesis represents the developmental connection between successful reproduction and recruitment. Detailed developmental descriptions remain particularly important for species whose artificial propagation, selective breeding, or conservation protocols are still being established. Wang et al. [20], for example, studied the embryogenesis, early larval morphology, and growth patterns of the tomato hind grouper *Cephalopholis sonnerati*, an emerging aquaculture species. This study provides a systematic characterization of *C. sonnerati* and establishes key baseline parameters, such as fertilization and hatching rates, cleavage patterns, size and characteristics of larvae, as well as growth performance, that are essential for standardizing cultivation protocols for this species.

A complementary conservation viewpoint is provided by Honji et al. [21], who described the embryonic and early larval development of the threatened Neotropical fish *Prochilodus vimboides*. The study established a species-specific developmental sequence encompassing zygote formation, cleavage, morula and blastula stages, gastrulation, organogenesis, hatching, and early yolk-sac absorption. In general, for threatened fish, developmental standards of this type extend beyond descriptive embryology: they support evaluation of embryo quality, identification of sensitive developmental period, optimization of hatchery procedures, and the improvement of *ex situ* conservation and restocking programs.

## 5. Integrating Reproductive Biology Across Aquaculture, Conservation, and Ecology

Taken together, the contributions to this Special Issue demonstrate that fish reproductive biology and embryogenesis are understood as an interconnected continuum extending from endocrine signaling and germ-cell development to spawning, embryonic ontogeny, recruitment, and population-level consequences. The studies include mechanistic physiology, reproductive biotechnology, broodstock management, developmental biology, fishery science, conservation, and invasion ecology, yet converge on a common principle: reproductive traits are highly context-dependent, and their biological meaning emerges from interactions among intrinsic physiological mechanisms, environmental conditions, and species-specific life histories.

In conclusion, this multidisciplinary perspective is particularly important at a time when aquatic organisms are increasingly exposed to rapid environmental and anthropogenic changes. Temperature shifts, altered hydrological regimes, habitat degradation, pollution, exploitation, and biological invasions can affect different stages of the reproductive cycle and early development, often with consequences that become evident only at later life stages or at the population level. Thus, future research should continue to combine endocrine, cellular, molecular, developmental, behavioral, and ecological approaches, with emphasis on longitudinal studies, comparative frameworks, and identifying developmental and reproductive periods of sensitivity. Finally, by integrating fundamental and applied research, the studies presented here expand current understanding of how fish reproduce, develop, and persist across diverse ecological settings. They also reinforce the importance of species-specific knowledge for the development of responsible aquaculture practices, effective fishery management, conservation programs, and broader assessments of population resilience. Alongside other Special Issues published in *Animals* that address related aspects of fish reproduction, development, physiology, and aquaculture [22,23,24,25,26,27,28,29,30,31], this collection illustrates the substantial progress currently being made in these fields. At the same time, the rapid expansion of literature reinforces the need for careful and critical evaluation of the scientific evidence underlying each contribution. The Guest Editors are therefore pleased to present this Special Issue and invite readers to explore the advances reported herein, which collectively contribute to a broader and more integrative understanding of fish reproductive biology and embryogenesis.
